# Development of an efficient in vitro micropropagation and biochemical profiling of *Salvia halophila*, an endemic Turkish sage

**DOI:** 10.1186/s12870-026-08152-2

**Published:** 2026-01-19

**Authors:** Münüre Tanur Erkoyuncu

**Affiliations:** Department of Field Crops, Faculty of Agriculture, Selçuk University, Konya, 42130 Türkiye

**Keywords:** Salvia halophila, Micropropagation, Basal medium, Cytokinins, Phenolic compounds, Antioxidant activity, Rooting, Acclimatization

## Abstract

**Background:**

*Salvia halophila* Hedge is an endemic sage species confined to the salt steppe ecosystems of Central Anatolia (Türkiye), particularly around the Lake Tuz Basin, and is classified as Endangered (EN) by the IUCN. Its narrow distribution, low seed germination rates, and habitat pressures threaten its survival and restrict the sustainable exploitation of its valuable phytochemicals, such as rosmarinic acid and other phenolics. In vitro micropropagation represents a strategic approach for producing genetically uniform and phytochemically rich plant material, thereby supporting both ex situ conservation and biotechnological applications.

**Results:**

Three germination strategies were compared. Chemical priming and direct gibberellic acid (GA₃) applications were largely ineffective, whereas in vitro germination on GA₃-enriched media produced viable seedlings. WPM medium supplemented with 0.5–1.0 mg L⁻¹ GA₃ achieved the highest germination rate (20%). The optimized sterilization treatment minimized fungal contamination (4.4%) while sustaining a high proportion of healthy shoots (73%). For shoot induction, MS medium consistently outperformed WPM, achieving 78% induction and 1.51 shoots per explant versus 50% and 0.86 on WPM. Among cytokinins, meta-topolin (mT) provided the most reliable results, ensuring 90–100% shoot induction with high shoot quality at 0.2–1.0 mg L⁻¹. Rooting experiments demonstrated the superiority of IBA over NAA, with 0.5 mg L⁻¹ IBA yielding 66% rooting and 77% acclimatization. Biochemical profiling showed that micropropagated plantlets accumulated higher phenolic contents (TPC = 15.9 mg GAE g⁻¹ DW) and stronger antioxidant capacity (IC₅₀=24.6 µg mL⁻¹) compared with seed-derived plants.

**Conclusion:**

The study successfully established a stage-specific and efficient micropropagation protocol for *S. halophila*. While WPM medium was more favorable for seed germination, MS medium supplemented with mT and IBA was optimal for shoot regeneration and rooting. The protocol not only enhances propagation efficiency but also increases secondary metabolite accumulation, providing a reliable platform for conservation, sustainable utilization, and potential commercial production of this endangered halophyte.

**Graphical abstract:**

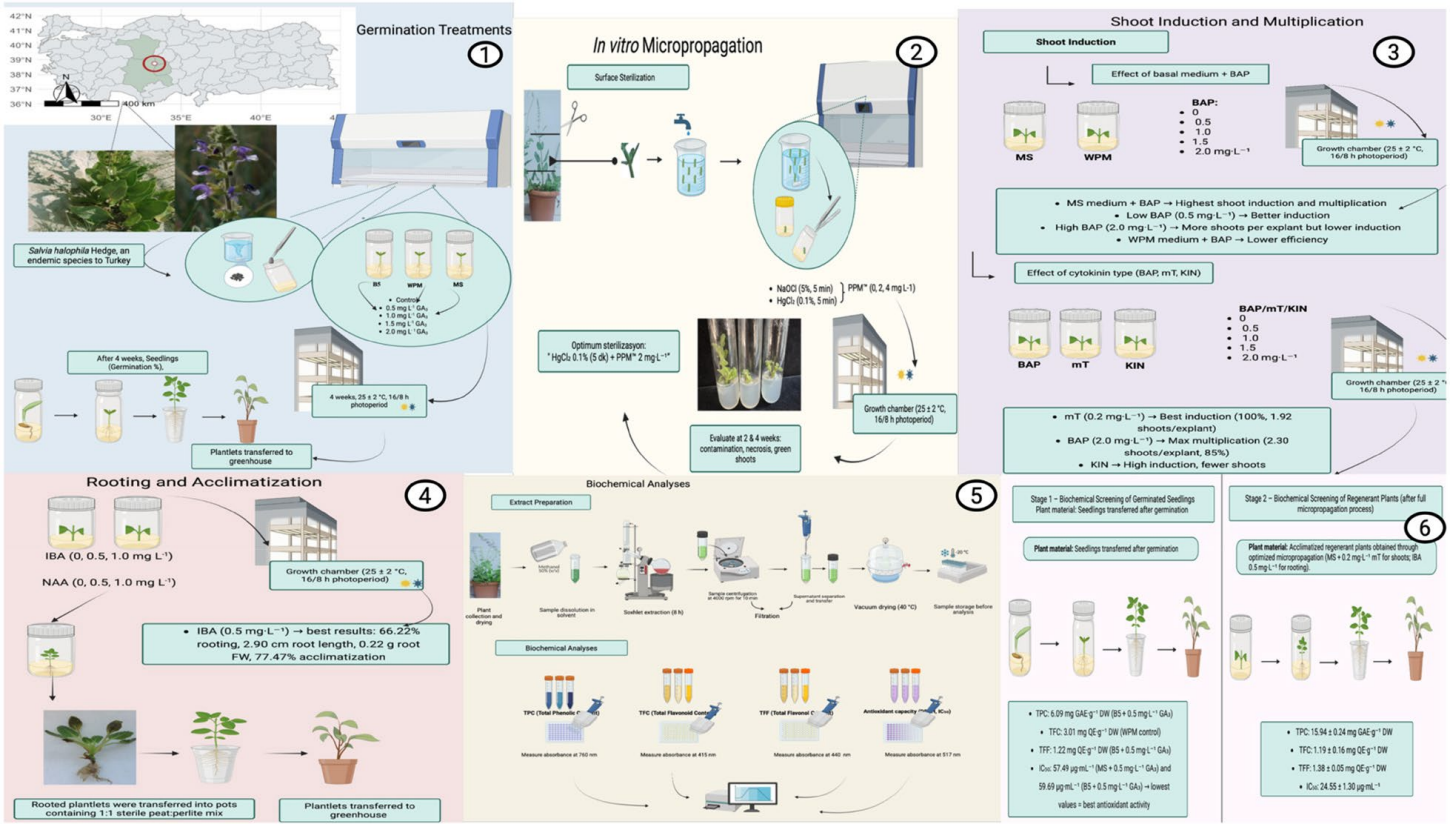

**Supplementary Information:**

The online version contains supplementary material available at 10.1186/s12870-026-08152-2.

## Introduction

 The Lamiaceae family is among the richest in the flora of Türkiye, with regard to species diversity and endemism. *Salvia*, the largest genus of this family, is represented by nearly 1,000 species worldwide [1] and by more than 100 taxa in Türkiye, more than half of which are endemic [2, 3]. Endemic *Salvia* species are of great interest not only for their ecological adaptations but also for their rich phytochemical composition, which underpins both their scientific value and their traditional uses. One of these endemic species, *Salvia halophila* Hedge, is naturally restricted to the salt steppe ecosystems of Central Anatolia (Türkiye), occurring in small populations particularly around the Lake Tuz Basin. According to the International Union for Conservation of Nature (IUCN), this species is classified as Endangered (EN) [4]. Its remarkable adaptation to extreme environmental conditions such as high salinity and drought makes it an ecologically significant plant [5]. Locally known as “Tuzlu Adaçayı” (Salt Sage), it has traditionally been used in the region as both a spice and an herbal tea [3, 6].

Phenolic acids and flavonoids commonly found in Lamiaceae species are of substantial pharmacological importance due to their strong antioxidant, antimicrobial, anti-inflammatory, and cytoprotective activities [7, 8]. In particular, bioactive compounds characteristic of the *Salvia* genus—such as rosmarinic acid and caffeic acid derivatives—are known for their biological functions including free-radical neutralization, reduction of oxidative damage, inhibition of microbial growth, and enhancement of cellular defense responses [9, 10]. In *Salvia* species, rosmarinic acid, caffeic acid, and their derivatives have been identified as major phenolic constituents, and these compounds have been reported to exhibit high antioxidant and antimicrobial activities [11–13]. Moreover, extracts of these species have shown strong antioxidant capacity in assays such as DPPH and ABTS and demonstrated significant antimicrobial activity particularly against Gram-positive bacteria [7, 10]. Consistent with these findings, *S. halophila* also contains a rich profile of phenolic compounds. Rosmarinic acid is the predominant phenolic constituent, while other identified compounds include caffeic acid, o-coumaric acid, gallic acid, p-hydroxybenzoic acid, luteolin-7-O-glucoside, and luteolin [6, 14]. Extracts of this species have demonstrated pronounced antioxidant and antimicrobial activities [5], highlighting its importance not only as a pharmacologically valuable species but also as a genetic resource of conservation priority.

Nevertheless, its restricted natural distribution, habitat pressures, and low seed germination rates—caused by seed dormancy and mucilage, which are common obstacles in other *Salvia* species [15–17]- pose serious threats to both the survival of *S. halophila* in nature and the sustainable use of its bioactive compounds. In species such as *S. halophila*, where natural regeneration depends primarily on seedling recruitment, low germination rates restrict the establishment of new individuals, slow down population turnover, and lead to an age-skewed population structure dominated by older plants. This situation is particularly problematic for narrowly distributed and small populations, as reduced recruitment ultimately decreases genetic diversity and increases vulnerability to environmental fluctuations [18, 19]. Similarly, genetic analyses conducted on *S. brachyodon* have shown that most new individuals originate from seeds, and that low germination and insufficient seedling establishment result in population structures dominated by older genotypes with diminished genetic diversity [19]. In small and fragmented populations, this process further accelerates genetic erosion and increases the risk of local extinction. Therefore, enhancing germination rates and promoting the recruitment of young individuals into the population are critical for the long-term sustainability of species such as *S. halophila*. In vitro micropropagation techniques provide a strategic solution for producing genetically uniform plant material in a short time, with high efficiency, and independent of environmental conditions [20]. Particularly for endemic species with limited seed propagation, micropropagation is valuable not only for conservation purposes but also for the multiplication of genotypes rich in secondary metabolites for integration into commercial production [21–23]. In addition, this technique prevents overharvesting from natural habitats, thereby contributing to the sustainability of wild populations, while ensuring the preservation and standardization of phytochemical contents under controlled conditions.

Previous in vitro micropropagation studies on closely related *Salvia* species have shown that shoot regeneration varies substantially depending on genotype, culture medium, and the type and concentration of growth regulators applied [24, 25]. For example, in *S. officinalis* and *S. tomentosa* the highest shoot multiplication rates were generally obtained with low concentrations of BA or mT, whereas higher cytokinin levels often resulted in hyperhydricity and other physiological abnormalities [20, 24, 25]. In addition, studies on species such as *S. miltiorrhiza*, *S. bulleyana*, and *S. sclarea* have demonstrated that growth regulators strongly influence not only shoot proliferation but also phenolic composition and antioxidant activity under in vitro conditions [22, 24, 26]. Despite the importance of such techniques, no reports are available on tissue culture studies of *S. halophila*. Therefore, the present study aimed to establish an efficient in vitro propagation protocol for this endemic and endangered species, providing scientific support for local conservation strategies and contributing to the sustainable utilization of its pharmacological potential.

## Materials and methods

### Plant material

*Salvia halophila* Hedge is an endemic species previously described in the flora of Türkiye. The seeds used in this study were kindly provided by Prof. Dr. Yüksel Kan (Selçuk University, Konya, Türkiye). Voucher specimens of *S. halophila* (Herbarium No: AEF 23649) were originally deposited in the Herbarium of the Faculty of Pharmacy, Ankara University (AEF), identified and confirmed by Prof. Dr. H. Duman and Dr. G. Yılmaz as reported by Orhan et al. [27]. To ensure traceability of the plant material, the locality and date of seed collection were also indicated: seeds of the endemic species were collected from Konya (Karakulluk–Eskil) in 2022.

### Surface sterilization and pretreatments

Since halophytic endemic species often exhibit strong dormancy constraints, several pretreatments were applied to enhance germination capacity. Prior to germination experiments, seeds were subjected to cold stratification by storage at 5 °C in darkness for 45 days [28], followed by surface sterilization in 5% (w/v) sodium hypochlorite (NaOCl) solution for 10 min, and subsequently rinsed three times with sterile double-distilled water (5 min each) [15, 22].

Previous studies on *Salvia* species have demonstrated that low germination rates are generally not caused by loss of seed viability but rather by physiological dormancy and the inhibitory effects of seed-coat mucilage on water uptake and gas exchange [29–31]. For this reason, many studies have not included a separate viability test prior to germination, as reduced germination is primarily attributed to dormant but viable seeds or to mucilage-related physical barriers.

To stimulate seed germination, three different approaches were employed:

### Chemical and hormonal priming treatments (KNO₃, HCl, NaCl, PEG600, GA₃)

After stratification and sterilization, seeds were subjected to chemical and hormonal priming treatments.

(i) Chemical/osmo-priming: Seeds were soaked for 24 h at 10 °C in KNO₃, HCl, NaCl, or PEG600 solutions at concentrations of 0.5, 1.0, 2.0, and 3.0%, while the control group was incubated in distilled water. The temperature of 10 °C was selected based on previous reports indicating that low-temperature hydration improves seed metabolic stability and germination performance in Salvia species [28]. In osmo priming and chemical priming applications, the pH of the solutions is generally used without adjustment, at their natural pH values. Several studies have reported this as a standard methodological approach, and no evidence has been presented indicating that pH modification improves priming efficiency [32, 33]. After priming, seeds were subjected to germination tests for 14 days under controlled conditions (25 ± 2 °C, 16/8 h photoperiod) [15, 34].
(ii) Hormonal priming with GA₃: Stratified and sterilized seeds were moistened with GA₃ solutions prepared at concentrations of 0 (distilled water), 250, 500, 1000, and 2000 mg L⁻¹. During the germination period, GA₃ solutions were applied every other day at a volume of 10 mL per Petri dish. Germination was monitored for 14 days under the same growth conditions [28, 35].

### In vitro germination on GA₃-supplemented media

Stratified and sterilized seeds were cultured on different basal media (MS [36], WPM [37], B5 [38]) supplemented with 0, 0.5, 1.0, or 2.0 mg L⁻¹ GA₃. Cultures were incubated under the same growth conditions [39, 40].

### Experimental design and seedling handling

Each treatment was carried out with four replicates, using 25 seeds per replicate. For subsequent experiments, only seedlings obtained from the third method (in vitro culture with GA₃-supplemented media) were evaluated. Germination was considered successful when the radicle reached approximately 2 mm in length. Germination percentages were recorded after four weeks of in vitro culture, whereas chemical and hormonal pretreatments were evaluated after 14 days. The germinated seedlings were subsequently maintained under the same growth conditions for further development and acclimatization. Once the seedlings reached an appropriate size, nodal explants were collected and used as explant sources for in vitro micropropagation experiments.

### In vitro micropropagation

#### Surface sterilization of nodal segments

Nodal segments obtained from acclimatized seedlings were used to initiate in vitro shoot cultures. At this stage, sterilization protocols were optimized to minimize both endogenous and exogenous contamination while maintaining explant viability. Two disinfectants were tested: sodium hypochlorite (NaOCl, 5%) [25] and mercuric chloride (HgCl₂, 0.1%) [26], each applied for 5 min. After each sterilization treatment, the explants were rinsed with sterile distilled water, three times for 5 min each. In addition, Plant Preservative Mixture™ (PPM™) was incorporated into the culture medium at concentrations of 0, 2, and 4 mg L⁻¹ to suppress endophytic contamination [41–43]. Explants were assessed on Day 14 (Week 1) and Day 28 (Week 2) for bacterial and fungal contamination, mortality, necrosis, and the emergence of green shoots. Each treatment was conducted with three replicates, with 15 nodal explants per replicate. The most effective sterilization protocol was subsequently adopted as the standard method for all micropropagation experiments.

### Shoot induction experiments

#### Effect of basal medium and BAP concentrations

For multiple shoot induction, single-node explants (1.0–1.5 cm in length), each containing one axillary bud and approximately 0.5–1.0 cm of internodal tissue, were excised from healthy and acclimatized plantlets. All regenerated shoots originated exclusively from pre-existing axillary buds located on the single-node explants, and no callus formation or adventitious shoot development was observed at any stage of the culture. Explants were then cultured on MS and WPM basal media supplemented with different concentrations of 6-benzylaminopurine (BAP) [0, 0.2, 0.5, 1.0, and 2.0 mg L⁻¹]. After five weeks of culture, the percentage of shoot induction and the mean number of shoots per explant (number) were recorded.

#### Effect of cytokinin types (BAP, mT, KIN)

To further optimize shoot multiplication, three different cytokinins—BAP, meta-Topolin (mT), and kinetin (KIN)—were tested in the most responsive basal medium at concentrations of 0, 0.2, 0.5, 1.0, and 2.0 mg L⁻¹. At the end of the culture period, shoot induction percentage (%) and mean shoot per explant (number) were measured. In addition to quantitative responses, the morphological quality of regenerated shoots (vigor, leaf size, and occurrence of vitrification) was also evaluated. Shoot vigor was qualitatively assessed based on overall shoot robustness, leaf size, and the absence of chlorosis or necrosis. Vitrification was evaluated qualitatively as the presence or absence of translucent, hyperhydrated tissues on leaves and stems.

#### Rooting and acclimatization

Regenerated shoots were transferred to MS medium supplemented with indole-3-butyric acid (IBA) or α-naphthaleneacetic acid (NAA) at concentrations of 0, 0.5, and 1.0 mg·L⁻¹ for rooting induction. After four weeks, rooting percentage (%), root length (cm), and root fresh weight (g) were determined. Rooted plantlets were initially transferred to a peat: perlite (1:1, v/v) mixture under high-humidity conditions in a growth chamber (25 °C, 16/8 h light/dark photoperiod). Plants were then gradually acclimatized to greenhouse conditions. The survival of acclimatized plants under *ex vitro* conditions was expressed as acclimatization success (%). Since the number of regenerated shoots varied among cytokinin treatments, the number of plantlets transferred to *ex vitro* conditions differed between treatments. Therefore, acclimatization success (%) was calculated for each treatment as: (number of surviving plantlets / number of plantlets transferred to *ex vitro* conditions) × 100.

All culture media were solidified with 0.8% (w/v) agar and supplemented with 3% (w/v) sucrose as the carbohydrate source. Media were sterilized by autoclaving at 121 °C for 20 min under 1.5 atm pressure. In vitro cultures were established with four replicates per treatment, each replicate consisting of five explants. Cultures were incubated in a controlled growth chamber (Sanyo MLR-351 H) for five weeks at 24 ± 2 °C, with a relative humidity of 65%, under a 16/8 h light/dark photoperiod and a light intensity of approximately 5 µmol m⁻² s⁻¹. All plant growth regulators used in this study were of analytical grade. GA₃, BAP, NAA, IBA, and KIN were purchased from Sigma-Aldrich (St. Louis, MO, USA), while meta-topolin (mT) was obtained from Duchefa Biochemie (Haarlem, The Netherlands). Sucrose, agar, and other medium-grade chemicals were supplied by Merck (Darmstadt, Germany).

### Biochemical analyses

#### Sample preparation and extraction

Biochemical analyses were performed both on plants germinated and acclimatized under different culture conditions and on regenerant plants obtained after completing the entire micropropagation process. The aerial parts of the plants were shade-dried at room temperature and ground into a fine powder using a mechanical grinder. For biochemical assays, plantlets that completed their development at the end of the culture period were collected simultaneously. Only healthy, fully developed, contamination-free plantlets of comparable size were selected for analysis. Subsequently, 1 g of powdered material was extracted in a Soxhlet apparatus with 10 mL of 50% methanol for 8 h [6]. Following extraction, the extracts were filtered and concentrated to dryness under vacuum at 40 °C.

A solvent-to-sample ratio of 1:10 (w/v) was employed for all Soxhlet extractions. Extraction yield (%) was not determined, as the aim of the extraction was to obtain sufficient extract for biochemical analyses rather than to compare extraction efficiency. All extracts were stored at − 20 °C until analysis.

#### Determination of phenolic compounds (TPC, TFC, TFF)

Total phenolic content was determined as gallic acid equivalents (GAE) per gram of dry weight [44]. For this, 100 µL of extract (4 mg mL⁻¹) was mixed with 6.0 mL distilled water and 500 µL undiluted Folin–Ciocalteu reagent. After 1 min, 1.5 mL of 20% (m/V) Na₂CO₃ solution was added, and the volume was made up to 10.0 mL with distilled water. Samples were incubated at 25 °C for 2 h, and absorbance was measured at 760 nm against a gallic acid calibration curve. Data were expressed as mean values of triplicate measurements (calibration range: 25–500 mg L⁻¹, R² = 0.996).

Total flavonoid and flavonol contents were expressed as quercetin equivalents (QE) in mg QE g⁻¹ dry weight [45]. For flavonoids, 1.0 mL of extract (4 mg mL⁻¹) was mixed with 1.0 mL of aluminum chloride solution (20 g L⁻¹ in ethanol) and diluted to 25.0 mL with ethanol. After incubation at 24 °C for 40 min, absorbance was measured at 415 nm. Blank samples were prepared by adding one drop of acetic acid to 1.0 mL extract (4 mg mL⁻¹) and diluting to 25.0 mL. Calibration curves were constructed with standard quercetin solutions following the same procedure. All measurements were performed in triplicate, and results were presented as mean values (calibration range: 25–500 mg L⁻¹, R² = 0.996).

For flavonols, 2.0 mL of extract (4 mg mL⁻¹) was mixed with 2.0 mL aluminum chloride (20 g L⁻¹) and 6.0 mL sodium acetate (50 g L⁻¹). After incubation at 20 °C for 2.5 h, absorbance was measured at 440 nm [45]. Quercetin calibration curves were prepared in the same way, and analyses were conducted in triplicate (calibration range: 25–500 mg L⁻¹, R² = 0.994).

#### Antioxidant activity (DPPH radical scavenging assay)

The antiradical activity of the extracts was assessed based on the radical-scavenging effect of the stable 1,1-diphenyl-2-picrylhydrazyl (DPPH) free radical, following the classical method of Blois [46] with modifications commonly applied in antioxidant screening studies Koleva et al., [47]. Extract solutions were prepared in the same solvent at different concentrations (1, 0.5, 0.25, and 0.1 mg mL⁻¹). A 50 µL aliquot of each extract was mixed with 450 µL Tris-HCl buffer and 1000 µL of 0.1 mM DPPH solution in methanol. After incubation at room temperature in the dark for 30 min, the decrease in absorbance was measured spectrophotometrically at 517 nm [5]. All assays were performed in triplicate, and results were expressed as mean values. The DPPH scavenging activity (%) was calculated using the following formula:

1 $$DPPH\:radical\:scavenging\:activity\:(\%)=((A_{a}-A_{s})/A_{a})\times100$$

Aₐ : Absorbance of the control.

Aₛ : Absorbance of the sample.

From the obtained data, IC₅₀ (Half Maximal Inhibitory Concentration, defined as the extract concentration required to scavenge 50% of DPPH radicals) values (µg mL⁻¹) were calculated by nonlinear regression analysis. Butylated hydroxytoluene (BHT) was used as the positive antioxidant standard, and the same DPPH procedure was applied to BHT solutions to generate standard calibration curves.

### Statistical analyses

All experimental data were subjected to analysis of variance (ANOVA) using JMP Pro 17 statistical software (SAS Institute Inc., Cary, NC, USA), with interaction effects evaluated according to the objectives and design of each experiment. Mean separations were performed with Tukey’s multiple range test at a significance level of *P* ≤ 0.05. All results were expressed as mean ± standard error (SE) based on at least three replicates, unless otherwise stated. Graphical representations were generated in GraphPad Prism (v10). For multivariate analyses, heatmaps and principal component analysis (PCA) were carried out in RStudio (2024.12.1) using the heatmaply, ggbiplot, and factoMineR packages. Before multivariate analysis, all biochemical variables were normalized using z-score standardization (mean-centering and scaling to unit variance).

## Results and discussion

### Seed germination approaches

Germination percentages were recorded after four weeks of in vitro culture. The effects of GA₃ concentrations and basal media on germination percentage are presented in Table 1. The highest germination rate (20%) was observed in WPM medium supplemented with 0.5 or 1.0 mg L⁻¹ GA₃. By comparison, germination frequencies on B5 and MS media remained very low and did not differ significantly. When averaged across treatments, WPM medium (13%) supported significantly higher germination than B5 (3%) and MS (2%) media (*P* < 0.01). GA₃ concentrations also exerted a significant main effect (*P* < 0.05); however, the medium × concentration interaction was not statistically significant.

**Table 1:** Effect of GA₃ concentrations and basal media on seed germination percentage

GA₃ (mg L⁻¹)	WPM	B5	MS	GA₃ Cons. mean
0	5	0	0	*1.67 ± 0.96* ^*B*^
0.2	10	5	5	*6.67 ± 0.96* ^*AB*^
0.5	20	10	5	*11.67 ± 2.55* ^*A*^
1.0	20	0	0	*6.67 ± 3.85* ^*AB*^
2.0	10	0	0	*3.33 ± 1.92* ^*AB*^
*Media mean*	*13.00 ± 0.15* ^*A*^	*3.00 ± 0.10* ^*B*^	*2.00 ± 0.06* ^*B*^	

Values are mean ± SE (*n* = 4). Two-way ANOVA and Tukey’s HSD test (*P* ≤ 0.05) were used. Different lowercase letters indicate interaction effects; uppercase letters indicate main factor effects

Chemical priming (i) and GA₃ treatments (ii) resulted in consistently low germination rates across all concentrations, preventing the generation of statistically meaningful data. Moreover, the few seeds that did germinate failed to progress beyond the cotyledon stage and exhibited no further development. In contrast, the third approach (iii)—in vitro culture on different basal media supplemented with GA₃—proved effective. This method yielded healthy seedlings that developed normally and subsequently served as nodal explant sources for micropropagation experiments. Seed dormancy and low germination rates are widely reported problems in *Salvia* species. These limitations are generally attributed to factors such as the presence of a mucilage layer on the seed coat, reduced embryo viability, and physiological dormancy [48]. Consequently, a range of pretreatments—including cold stratification, chemical priming, and hormone applications—have been tested across different species. However, their effectiveness has varied considerably depending on the species, treatment dose, and environmental conditions [34, 49–52]. In our study, chemical priming and direct GA₃ applications in *S. halophila* resulted in low germination rates, further highlighting the complexity of germination biology within the *Salvia* genus and the pronounced interspecific differences [53, 54]. Similar trends have been reported in other species such as *S. microstegia*,* S. hispanica* and *S. officinalis*, where classical chemical applications and stress conditions had only a limited effect on germination [55–57]. By contrast, applications involving the supplementation of GA₃ into in vitro culture media were successful, resulting in the production of healthy seedlings. In particular, the WPM medium provided the highest germination rate compared to other media, showing strong parallels with findings reported for species such as *S. sclarea*, *S. macrosiphon*, and *S. plebeia* [58–60]. This superior performance of WPM may be attributed to its lower total salt concentration and more balanced mineral formulation. As *S. halophila* is a halophytic species naturally adapted to low-nutrient, saline steppe soils, a medium with lower ionic strength may better mimic its ecological conditions, facilitating water uptake and embryo activation. This could explain why WPM supported higher germination than MS or B5, both of which contain higher macronutrient levels. Moreover, the use of GA₃ at different concentrations has been shown to play a critical role in overcoming dormancy and promoting seedling development, a finding also corroborated in studies on *S. hispanica* and *S. miltiorrhiza* [26, 61, 62]. The findings indicate that germination success in *Salvia* species depends not only on hormone applications but also on the mineral and vitamin composition of the medium. Thus, GA₃-enriched culture media provide an effective strategy for overcoming dormancy barriers and promoting healthy seedling development.

In *S. halophila*, seed germination and early seedling development are strongly shaped by ecological adaptations associated with halophytism and physiological dormancy. Germination in halophytes is frequently constrained by low-temperature requirements, seed-coat–related limitations to water uptake, and mechanisms of physiological dormancy that maintain ionic balance under saline conditions [63, 64]. Although halophytic species such as *S. halophila* tolerate high salinity at later developmental stages, the germination phase is typically more sensitive to salt stress than established seedlings or adult plants [65]. Physiological dormancy in halophytes functions as a survival strategy, delaying germination until optimal environmental conditions occur [64, 66]. Accordingly, the enhanced germination observed following cold stratification and GA₃ application reflects their ability to overcome this dormancy and activate early metabolic processes [67]. GA₃, in particular, is known to stimulate germination in physiologically dormant seeds, consistent with findings reported for *S. halophila* and related halophytic taxa [68]. Similarly, cold stratification increases seed-coat permeability, promotes water uptake, and triggers embryo activation [67]. In addition, osmo-priming and hormonal priming alleviate the sensitivity of the germination stage to salt stress by promoting water absorption, metabolic activation, and early embryo growth [69]. The effectiveness of these treatments in overcoming ecological constraints under in vitro conditions supports the conclusion that the germination behavior observed in *S. halophila* is consistent with its natural ecology [70].

### Phenolic content and antioxidant capacity of in vitro–germinated seedlings under different culture media and GA₃ treatments

Four-week-old in vitro–germinated seedlings were acclimatized and subsequently transferred to *ex vitro* conditions. All seedlings derived from WPM medium, regardless of GA₃ concentration, were successfully acclimatized, whereas in B5 and MS media only those obtained with 0.5 mg L⁻¹ GA₃ survived under external conditions. Although no quantitative data were recorded for acclimatization, the adapted seedlings were analyzed after eight weeks for their total phenolic (TPC), flavonoid (TFC), and flavonol (TFF) contents, as well as antioxidant activity. In this way, the biochemical composition of plantlets intended for subsequent micropropagation experiments was characterized in relation to the effects of culture medium and GA₃ supplementation.

As shown in Table 2, the phenolic and antioxidant profiles observed in *S. halophila* are broadly in agreement with those reported for related *Salvia* species. The TPC values obtained in this study (2.54–6.09 mg GAE g⁻¹ DW) are comparable to those reported for *S. officinalis* and S. *fruticosa* (4–10 mg GAE g⁻¹ DW), although wider ranges (2–184 mg GAE g⁻¹ DW) have been documented depending on extraction method, geographic origin, and environmental conditions [71–73]. In contrast, species such as *S. plebeia* generally exhibit lower TPC levels (2–4 mg GAE g⁻¹ DW) [74]. Likewise, the highest TFC value recorded in Table 2 (3.01 mg QE g⁻¹ DW) is consistent with or slightly higher than those reported for *S. sclarea* and *S. hispanica* (1.5–2.8 mg QE g⁻¹ DW) [71, 73]. Similarly, the TFF levels presented in Table 2 (0.80–1.22 mg QE g⁻¹ DW) closely match the flavonol ranges reported for *S. officinalis* and *S. macrochlamys* (0.6–1.4 mg QE g⁻¹ DW) [75]. The IC₅₀ values shown in Table 2 (57–68 µg mL⁻¹) also fall within the antioxidant activity ranges previously reported for *S. fruticosa* and *S. officinalis* (55–80 µg mL⁻¹). Taken together, Table 2 demonstrates that the phenolic and antioxidant characteristics of *in vitro–*derived *S. halophila* plantlets are largely comparable to those of closely related *Salvia* species, while highlighting the substantial influence of medium composition and GA₃ supplementation on secondary metabolite accumulation.

**Table 2:** Effects of culture medium and GA₃ treatment combinations on TPC, TFC, TFF and IC₅₀

Treatment	TPC (mg GAE g⁻¹ DW)	TFC (mg QE g⁻¹ DW)	TFF (mg QE g⁻¹ DW)	IC₅₀ (µg mL⁻¹)
B5-0.5 mg L⁻¹ GA₃	6.09 ± 0.03ᵃ	1.54 ± 0.19ᵇ	1.22 ± 0.01ᵃ	59.69 ± 0.39ᶜ
MS-0.5 mg L⁻¹ GA₃	5.40 ± 0.22ᵃᵇ	0.74 ± 0.10ᵇᶜ	0.66 ± 0.00ᶜᵈ	57.49 ± 0.34ᶜ
WPM- Control	4.68 ± 0.12ᵇᶜ	3.01 ± 0.19ᵃ	0.77 ± 0.01ᶜ	63.35 ± 0.55ᵇ
WPM-0.2 mg L⁻¹ GA₃	4.17 ± 0.03ᶜᵈ	0.31 ± 0.03ᶜ	0.71 ± 0.02ᶜᵈ	66.05 ± 0.65ᵃᵇ
WPM-0.5 mg L⁻¹ GA₃	3.60 ± 0.06ᵈ	0.50 ± 0.05ᶜ	0.66 ± 0.03ᶜᵈ	65.45 ± 0.49ᵃᵇ
WPM-1.0 mg L⁻¹ GA₃	5.16 ± 0.03ᵇ	0.82 ± 0.01ᵇᶜ	0.98 ± 0.03ᵇ	65.41 ± 0.23ᵃᵇ
WPM-2.0 mg L⁻¹ GA₃	2.54 ± 0.08ᵉ	0.06 ± 0.03ᶜ	0.54 ± 0.03ᵈ	68.60 ± 0.21ᵃ

Values represent mean ± SE (*n* = 3). Treatments represent specific combinations of basal media and GA₃ concentrations and were analyzed using one-way ANOVA followed by Tukey’s HSD test (*P* ≤ 0.05). Different lowercase letters indicate significant differences among treatment means

### Multivariate analysis of germination and biochemical traits

**Fig. 1:**
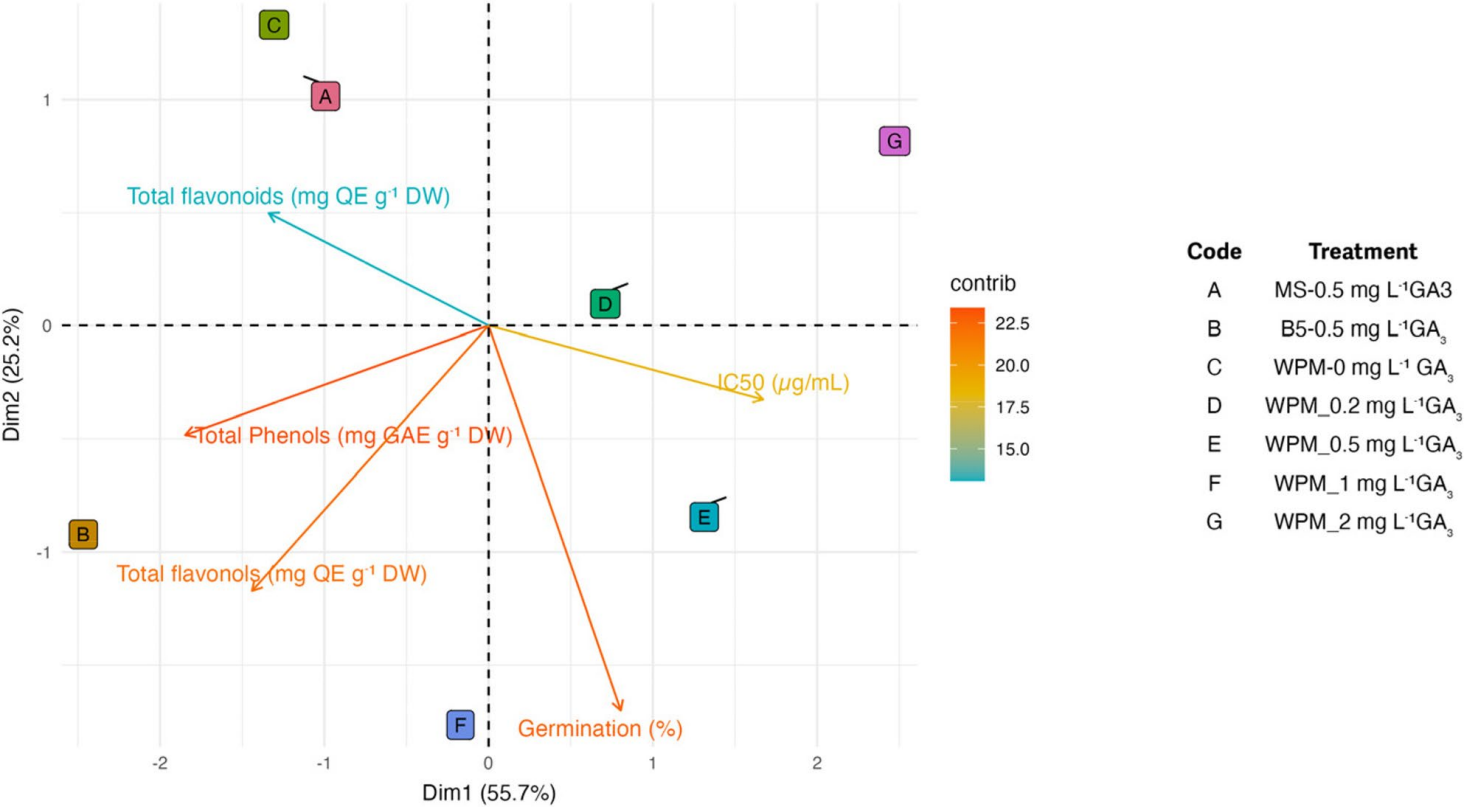
PCA biplot of germination percentage, phenolic contents (TPC, TFC, TFF), and antioxidant activity (IC₅₀) of *S. halophila* seedlings cultured on different basal media supplemented with GA₃

Principal component analysis (PCA) explained 80.9% of the total variance (Dim1: 55.7%, Dim2: 25.2%) (Fig. 1). The PCA biplot revealed two major axes explaining the variation among the biochemical and physiological variables. Dim1 (55.7%) represented the primary separation between phenolic-related variables and parameters associated with lower antioxidant capacity. Total phenolic content (TPC), total flavonols (TFF), and total flavonoids (TFC) were all oriented toward the negative side of Dim1, indicating a strong positive correlation among these phenolic components. However, TFC showed a distinct positive loading on Dim2, separating it from TFF and TPC and suggesting that flavonoids contribute an additional independent source of variation.

In contrast, IC₅₀ and germination percentage were positioned on the positive side of Dim1, demonstrating an inverse relationship with TPC, TFF, and TFC. IC₅₀ oriented toward the upper-right quadrant, while germination percentage loaded toward the lower-right quadrant, reflecting that these two variables share the same Dim1 direction but differ along Dim2.

Treatment groupings on the biplot were consistent with these variable associations. MS medium supplemented with 0.5 mg L⁻¹ GA₃ (A), GA₃-free WPM medium (C), and WPM with 1 mg L⁻¹ GA₃ (F) clustered on the left side of the biplot, corresponding to higher levels of phenolic constituents. In contrast, WPM medium supplemented with 0.2, 0.5, and 2 mg L⁻¹ GA₃ (D, E, and G) appeared on the right side, closely associated with higher IC₅₀ values and increased germination percentage. These findings clearly demonstrate that GA₃ concentration and medium type modulate the main biochemical axes governing phenolic accumulation and antioxidant capacity in in vitro-germinated seedlings.

The PCA results demonstrated that different basal media and GA₃ treatments caused both statistically and biologically meaningful differentiation in phenolic compound accumulation, antioxidant activity, and germination behavior of *S. halophila* seedlings.

Phenolic compounds (TPC, TFF, and TFC) clustered together on the negative side of Dim1, indicating that these metabolites are synthesized under a shared regulatory mechanism during early seedling development. The common precursors of the phenylpropanoid pathway (e.g., phenylalanine) and the rapid activation of this pathway under oxidative stress conditions explain the simultaneous increase in phenolic and flavonoid compounds. In vitro–germinated tissues frequently produce elevated levels of reactive oxygen species (ROS) in response to environmental factors such as light exposure, nutrient availability, and hormonal signals, which in turn triggers the activation of phenolic metabolism as a protective defense mechanism [76, 77].

The positioning of the IC₅₀ variable on the positive side of Dim1 indicates its inverse relationship with phenolic compounds, which is a natural consequence of the radical-scavenging properties of phenolic metabolites. The hydroxyl groups of phenolic compounds can donate electrons or hydrogen atoms to neutralize reactive oxygen species (ROS); therefore, as phenolic accumulation increases, IC₅₀ values decrease. This inverse association is fully consistent with the opposite loading patterns observed along the PCA axes [78, 79].

The separation of germination percentage from phenolic variables, with germination loading on the positive side of Dim1, is biologically meaningful in terms of metabolic priorities. During germination, metabolic energy is primarily allocated to cell expansion, enzymatic mobilization, and hormonal signaling. Consequently, carbon skeletons are directed toward growth-related pathways rather than secondary metabolite biosynthesis, which may limit phenolic accumulation. This pattern is particularly evident under GA₃ treatments, as GA₃ accelerates germination through endosperm weakening and the activation of hydrolytic enzymes, potentially suppressing certain branches of phenolic metabolism [80, 81].

The separation of TFC from TPC and TFF along Dim2 suggests that flavonoid subclasses are more responsive to culture medium composition and hormonal signaling. Flavonoids represent a more specific branch of the phenolic pathway, and their biosynthesis can respond more rapidly to environmental cues such as light or to growth regulators like GA₃. Therefore, the distinct loading of flavonoids on Dim2 likely reflects biochemical responses specific to variations in GA₃ concentration [82–84].

Different basal media and GA₃ concentrations were found to exert significant effects on the phenolic compound production and antioxidant capacity of *S. halophila*. Notably, the highest TPC level was obtained in B5 medium supplemented with a low concentration of GA₃, whereas WPM and B5 media were superior in terms of flavonoid accumulation. Comparable patterns have been reported in other *Salvia* species and medicinal plants, where the composition of the culture medium strongly influenced the metabolic profile [58, 85–87]. Specifically, phosphate concentration, pH, and mineral composition of the medium have been shown to directly regulate phenolic and flavonoid production.

Plant growth regulators (PGRs) also play an important role in modulating secondary metabolism [88]. Auxins, cytokinins, and salicylic acid have been demonstrated to stimulate phenolic accumulation and antioxidant activity in different *Salvia* species. For instance, a combination of 2,4-D and BAP enhanced phenolic content and antioxidant activity in *S. tebesana* [89], while synthetic cytokinins such as CPPU (N-(2-chloro-4-pyridyl)-N′-phenylurea) promoted phenolic production in *S. bulleyana* [61]. Our findings further confirm that GA₃ at lower concentrations can stimulate phenolic accumulation, whereas higher doses may exert inhibitory effects. Taken together, these results highlight the pivotal role of medium composition and PGR interactions in shaping secondary metabolite biosynthesis, thereby providing new insights into the biotechnological potential of *S. halophila*.

### In vitro micropropagation

#### Surface sterilization of nodal segments

Table 3 presents the effects of different disinfectant types, PPM™ concentrations, and culture duration on contamination, viability, and regeneration parameters. By the second week, fungal contamination was highest under NaOCl treatment (35.56%). The addition of 2 mg L⁻¹ PPM™ to the medium reduced this rate to 17.78%, while the proportion of green shoots increased to 66.67%. In contrast, HgCl₂ treatments generally showed lower contamination levels, and in the presence of 2 mg L⁻¹ PPM™, fungal contamination decreased to as low as 4.44%, with healthy shoot development reaching 73.33%.

**Table 3:** Effects of disinfectant types, PPM™ concentrations, and culture time on contamination, survival, and regeneration parameters of *S. halophila* nodal cultures

Time	Disinfectant (%)	PPM™ (mg L⁻¹)	Bacterial (%)	Fungal (%)	Mortality (%)	Green shoots (%)	Necrosis (%)
1st week (14 days)	NaOCl	0	11.11 ± 1.28ᵃᵇ	35.56 ± 1.28ᵃ	13.33 ± 0.00	37.78 ± 1.28ᵈᵉ	2.22 ± 1.28ᵃᵇ
		2	2.22 ± 1.28ᶜᵈ	17.78 ± 1.28ᵇ	13.33 ± 0.00	66.67 ± 0.00ᵃᵇ	0.00 ± 0.00ᵇ
		4	4.44 ± 1.28ᵇᵈ	13.33 ± 0.00ᵇᶜ	24.44 ± 1.28	60.00 ± 0.00ᵃᶜ	0.00 ± 0.00^b^
	HgCl₂	0	8.89 ± 1.28ᵃᶜ	11.11 ± 1.28ᵇᶜ	31.11 ± 1.28	48.89 ± 1.28ᵇᵉ	0.00 ± 0.00ᵇ
		2	0.00 ± 0.00ᵈ	4.44 ± 1.28ᶜ	22.22 ± 1.28	73.33 ± 0.00ᵃ	0.00 ± 0.00ᵇ
		4	0.00 ± 0.00ᵈ	6.67 ± 0.00ᶜ	26.67 ± 2.57	64.44 ± 1.28ᵃᵇ	0.00 ± 0.00ᵇ
2nd week (28 days)	NaOCl	0	15.56 ± 1.28ᵇᵈ	37.78 ± 1.28ᵃ	11.11 ± 1.28	33.33 ± 0.00ᵉ	2.22 ± 1.28ᵃᵇ
		2	11.11 ± 1.28ᶜᵈ	35.56 ± 2.57ᵃ	8.89 ± 1.28	37.78 ± 3.39ᵈᵉ	6.67 ± 0.00ᵃ
		4	4.44 ± 1.28ᵈ	13.33 ± 0.00ᵇᶜ	17.78 ± 1.28	62.22 ± 4.63ᵃᶜ	2.22 ± 1.28ᵃᵇ
	HgCl₂	0	15.56 ± 1.28ᵈ	11.11 ± 1.28ᵇᶜ	22.22 ± 1.28	42.22 ± 5.13ᶜᵉ	0.00 ± 0.00ᵇ
		2	0.00 ± 0.00ᵈ	6.67 ± 0.00ᶜ	24.44 ± 1.28	68.89 ± 1.28ᵃᵇ	0.00 ± 0.00ᵇ
		4	0.00 ± 0.00ᵈ	6.67 ± 0.00ᶜ	31.11 ± 4.63	57.78 ± 1.28ᵃᵈ	4.44 ± 1.28ᵃᵇ

Values are mean ± SE (*n* = 3). Three-way ANOVA and Tukey’s HSD test (*P* ≤ 0.05) were used. Different lowercase letters within each column indicate significant differences among Time × Disinfectant × PPM treatment combinations. Mortality (%) was not significantly affected by Time × Disinfectant × PPM treatments (*P* > 0.05); therefore, no superscript letters are shown in this column

At the fourth week, fungal contamination in NaOCl treatments further increased to 37.78%. Conversely, the combination of HgCl₂ + 2 mg L⁻¹ PPM™ completely eliminated bacterial contamination and maintained a high proportion of green shoots (68.89%). Mortality rates did not differ significantly among treatments, and necrosis was observed only in some NaOCl applications.

**Fig. 2:**
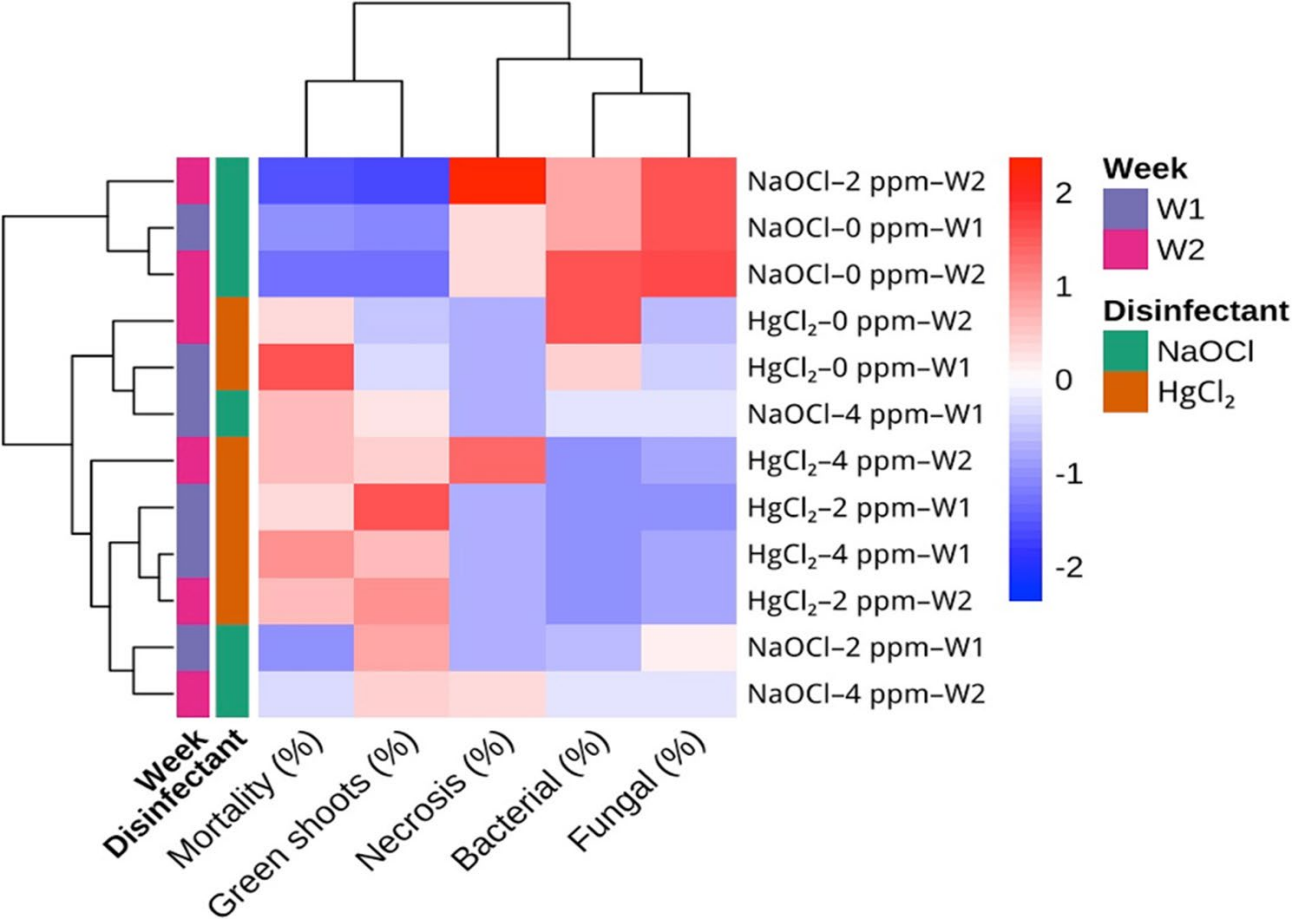
Heatmap of disinfectant effects on mortality, regeneration, and contamination parameters in *S. halophila* nodal explants. The heatmap with hierarchical clustering shows the effects of NaOCl and HgCl₂ at different concentrations on mortality (%), green shoot formation (%), necrosis (%), bacterial (%), and fungal (%) contamination. Week 1 (14 days after culture initiation) and Week 2 (28 days after culture initiation). The heatmap displays z-score–scaled values for each variable, where red indicates higher relative values, blue indicates lower relative values, and white represents the mean. Scaling was performed per variable to enable comparison of relative trends across treatments

The heatmap (Fig. 2) provided a multivariate overview of the interactions among disinfectant and PPM™ treatments. Bacterial and fungal contamination clustered inversely with green shoot percentage, whereas NaOCl treatments were associated with higher mortality and necrosis. In contrast, HgCl₂ + 2 mg L⁻¹ PPM™ grouped independently, characterized by minimal contamination and enhanced shoot regeneration. This multivariate visual analysis supported the trends observed in the tabulated data.

The present study revealed that sterilization efficiency in *S. halophila* nodal explants varied considerably depending on the disinfectant used and the inclusion of supplementary biocides. NaOCl alone was unable to completely suppress contamination, with fungal incidence reaching 37.78% by the fourth week. These findings indicate that NaOCl is effective against surface microorganisms but insufficient for controlling endophytic contamination, consistent with reports from other woody or pubescent species where NaOCl eliminated epiphytes but failed to eradicate internal bacteria [43, 90]. HgCl₂ treatments reduced both bacterial and fungal contamination more effectively; however, they were associated with mortality rates between 22 and 31%, reflecting its strong disinfectant action but also notable phytotoxicity. Literature reports confirm that while HgCl₂ is a powerful sterilant, its concentration and exposure duration must be carefully optimized to avoid tissue damage [91]. Both sodium hypochlorite and mercuric chloride are effective in controlling epiphytic microorganisms yet remain inadequate against endophytic contaminants. Consequently, the use of broad-spectrum biocides such as PPM™ has been recommended to address endophytic contamination in explants [92, 93]. Supporting this, the combination of HgCl₂ with a low dose of PPM™ (2 mg L⁻¹) not only minimized fungal contamination to 4.44% but also achieved the highest rate of healthy shoot development (73.33%). Similar outcomes have been reported in other plant species, where PPM™ reduced HgCl₂ toxicity while enhancing its disinfectant efficiency. By contrast, NaOCl treatments, though partially effective at the initial stages, were unable to prevent contamination resurgence in long-term cultures, where fungal growth and necrosis reappeared. Even when combined with PPM™, NaOCl did not fully eliminate contamination in highly infected material [43, 94, 95].

Taken together, these results highlight HgCl₂ + low-dose PPM™ as the most effective sterilization approach for *S. halophila*. This combination ensured both low contamination rates and high explant viability, offering a reliable foundation for sustainable in vitro cultures and subsequent micropropagation steps. Although the use of HgCl₂ combined with 4 mg L⁻¹ PPM™ resulted in a slightly lower contamination rate than the 2 mg L⁻¹ treatment, it did not improve the proportion of healthy or viable explants. Instead, a mild reduction in explant survival and early shoot growth was observed, indicating potential phytotoxicity of higher PPM™ concentrations. Previous studies also report that elevated PPM™ levels may suppress tissue vigor and increase physiological stress in delicate explants [96, 97]. Moreover, doubling the PPM™ concentration substantially increases the operational cost without yielding additional benefit. For these reasons, HgCl₂ + 2 mg L⁻¹ PPM™ was selected as the most effective and practical combination for routine micropropagation.

### Shoot induction experiments

#### Effect of basal medium and BAP concentrations

**Fig. 3:**
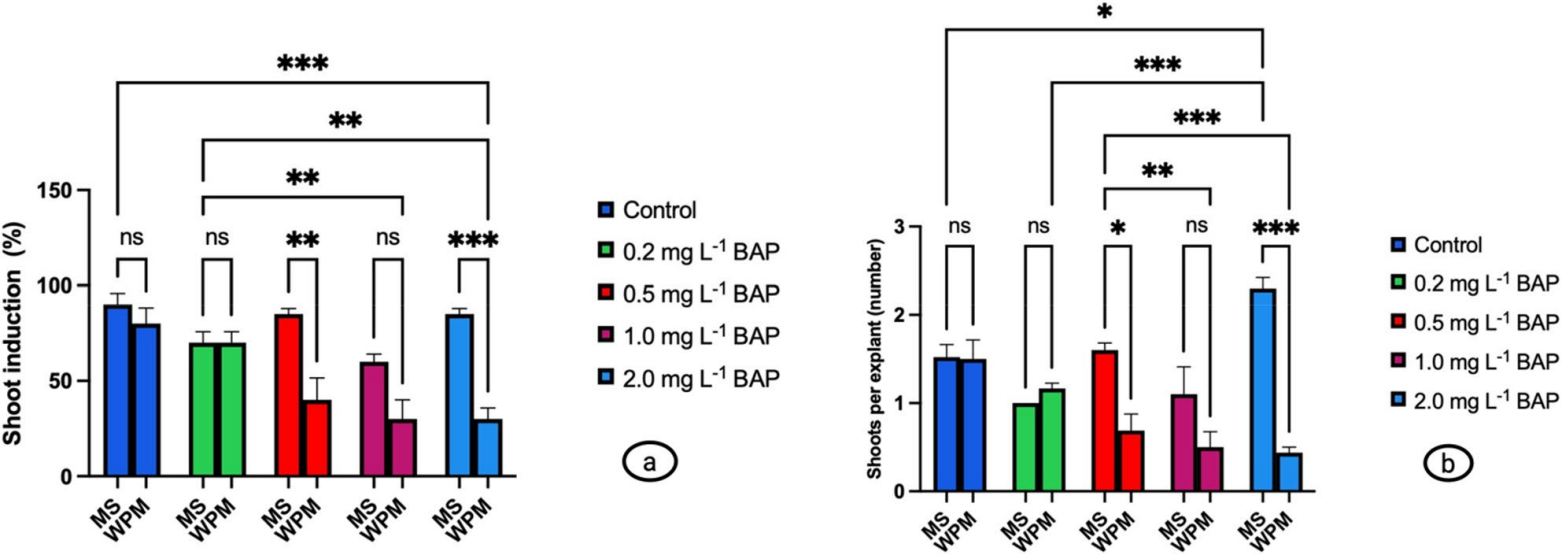
Effect of basal medium (MS and WPM) and BAP concentrations on shoot induction and shoot multiplication of *S. halophila* nodal explants. **a** Shoot induction percentage; (**b**) Number of shoots per explant. Values are mean ± SE (*n* = 4). Statistical differences were determined by two-way ANOVA followed by Tukey’s HSD test (*P ≤ 0.05, **P ≤ 0.01, ***P ≤ 0.001)

The shoot induction percentages and the number of shoots per explant obtained from *S. halophila* nodal explants cultured on MS and WPM media with different BAP concentrations are presented in Table 4; Fig. 3. According to the two-way ANOVA, medium, BAP concentration, and their interaction (medium types × BAP) had statistically significant effects on both shoot induction (%) and the number of shoots per explant (*P* < 0.01). Based on overall means, MS medium yielded higher shoot induction (78.0%) and shoot number per explant (1.51) compared to WPM (50.0%; 0.86 shoots/explant).

**Table 4:** Shoot induction percentage and number of shoots per explant of *S. halophila* cultured on MS and WPM media at different BAP concentrations

BAP (mg L⁻¹)	Shoot induction (%)		Shoots per explant (Number)		
	MS	WPM	MS		WPM
0.0	90.00 ± 2.89ᵃ	80.00 ± 4.08ᵃ	1.52 ± 0.07ᵇ		1.50 ± 0. 11ᵇ
0.2	70.00 ± 2.89ᵃᵇ	70.00 ± 2.89ᵃᵇ	1.00 ± 0.00ᵇᶜ		1.17 ± 0.03ᵇᶜ
0.5	85.00 ± 1.44ᵃ	40.00 ± 5.77ᵇᶜ	1.60 ± 0.04ᵃᵇ		0.69 ± 0.09ᶜ
1.0	60.00 ± 2.04ᵃᶜ	30.00 ± 5.00ᶜ	1.10 ± 0.16ᵇᶜ		0.50 ± 0.09ᶜ
2.0	85.00 ± 1.44ᵃ	30.00 ± 2.89ᶜ	2.30 ± 0.06ᵃ		0.44 ± 0.03ᶜ
*Overall mean*	*78.00ᴬ*	*50.00ᴮ*	*1.51ᴬ*		*0.86ᴮ*

Values are mean ± SE (*n* = 4). Two-way ANOVA and Tukey’s HSD test (*P* ≤ 0.05) were used. Lowercase letters indicate significant differences among BAP × medium interaction means; uppercase letters indicate main factor differences between MS and WPM media

On MS medium, the highest shoot induction was recorded in the control (90.0 ± 2.89%) and at 0.5 mg L⁻¹ BAP (85.0 ± 1.44%). The maximum number of shoots per explant was observed at 2.0 mg L⁻¹ BAP (2.30 ± 0.06), which was statistically different from all other treatments. On WPM medium, the highest induction rate was obtained in the control group (80.0 ± 4.08%); however, increasing BAP concentrations markedly reduced induction, dropping to as low as 30.0 ± 2.89% at 2.0 mg L⁻¹ BAP.

The graphical analysis presented in Fig. 3 clearly demonstrates the dose-dependent interaction between the basal medium and BAP concentration. Overall, MS medium performed better than WPM, particularly at 0.5 and 2.0 mg L⁻¹ BAP, where shoot induction and shoot number were significantly higher (*P ≤ 0.05, **P ≤ 0.01, *P ≤ 0.001). In contrast, differences between the two media were not statistically significant at 0.2 and 1.0 mg L⁻¹ (ns). Furthermore, a general trend was observed whereby lower BAP concentrations favored shoot induction, whereas higher concentrations promoted shoot multiplication. These results indicate that the response of S. halophila depends on both the basal medium and the specific BAP dose applied.

The higher shoot induction (78% vs. 50%) and shoot number per explant (1.51 vs. 0.86) observed on MS medium compared to WPM suggests that the mineral and vitamin composition of MS medium is more suitable for promoting cell division and meristematic activity. The relatively higher concentrations of macronutrients such as nitrogen and potassium, as well as the balanced supply of micronutrients, may provide a more favorable physiological environment for explant growth. Indeed, the superiority of MS medium in enhancing shoot induction has also been reported in several *Salvia* species, including *S. officinalis*, *S. guaranitica*, *S. santolinifolia*, *S. viridis*, *S. albimaculata*, *S. chrysophylla*, *S. ephratica* and *S. nydeggeri* where MS was found to stimulate both shoot initiation and proliferation more effectively than alternative basal media [98–102]. Similarly, in various medicinal and aromatic plants, MS medium has been reported to support higher morphogenetic responses in terms of both shoot induction and rooting compared to WPM or B5 [103–105]. This trend underscores the broader applicability of MS medium as a standard basal medium across a wide range of species, reinforcing its importance in propagation studies.

In MS medium, high shoot induction rates (85–90%) observed in the control and at 0.5 mg L⁻¹ BAP indicate that low BAP concentrations promote shoot initiation. In contrast, the maximum number of shoots per explant was recorded at 2.0 mg L⁻¹ BAP (2.30 shoots/explant), suggesting that higher hormone levels may reduce induction frequency while enhancing shoot multiplication capacity. This pattern reflects a hormetic effect, whereby BAP stimulates shoot initiation at lower concentrations and promotes shoot proliferation at higher concentrations, as also reported in the literature [106, 107]. Similar responses have been documented in different *Salvia* species, including *S. bulleyana*, *S. tebesana*, and *S. tomentosa* [25, 61, 89].

In WPM medium, although 80% shoot induction was achieved in the control group, increasing BAP concentrations reduced the induction rate to as low as 30%. This suggests that the salt composition and mineral balance of WPM may interact negatively with high cytokinin levels. Similarly, reports indicate that elevated cytokinin concentrations in WPM limit shoot induction not only in woody species but also in *Salvia* species, and this trend is supported in the literature [104, 108, 109].

Therefore, for *S. halophila*, the most favorable conditions for shoot induction were achieved on MS medium with low to moderate BAP concentrations, whereas higher BAP levels could be applied to maximize the number of shoots per explant. These findings highlight the dose-dependent variability in the interaction between medium composition and hormone concentration, underscoring the importance of species-specific protocol optimization. In this study, while seed germination was higher on WPM medium, shoot induction from nodal explants was more successful on MS medium, which may be attributed to the distinct metabolic requirements of these two developmental processes. Due to its low salt content and balanced ionic composition, WPM medium provides favorable conditions for germination physiology, particularly in species with high stress adaptation, by maintaining embryo viability and facilitating water uptake [39, 110–112]. In contrast, the higher nitrate and ammonium content of MS medium stimulates rapid cell division as well as protein and nucleic acid synthesis, thereby supporting shoot induction [107, 113]. Indeed, germination largely depends on reserve mobilization and energy utilization and thus does not require high mineral concentrations, whereas shoot regeneration demands active cell proliferation. Studies conducted within the *Salvia* genus demonstrate that germination success is strongly dependent on the rapid and efficient mobilization of stored reserves. In *S. officinalis* and closely related species, the hydrolysis of carbohydrate and lipid reserves through the activation of hydrolase enzymes such as amylases and lipases has been identified as a key determinant of early germination performance [114, 115]. Similarly, in *S. hispanica*, pronounced mobilization of lipid and protein reserves and extensive molecular reorganization occur during germination, processes essential for early seedling establishment [114]. The hydrolysis of stored reserves is largely regulated by plant hormones—particularly gibberellins—and is directly associated with high seed vigor [114, 116]. Conversely, under suboptimal conditions such as salinity or drought stress, reduced hydrolase activity and impaired reserve mobilization lead to delayed germination and lower germination percentages [117, 118]. Seeds with high vigor can achieve faster and more efficient reserve mobilization even under stress, resulting in superior germination performance [117].

Furthermore, the halophytic nature of *S. halophila* and its natural habitat characterized by low-nutrient and saline conditions may have contributed to the adaptation of its seed physiology to low-ion media such as WPM. However, under controlled tissue culture conditions, the regeneration of nodal explants responded more favorably to MS medium with its higher mineral content. Therefore, this contrasting response observed at different developmental stages reflects the interaction between medium composition and species-specific physiological adaptations, indicating that germination and shoot regeneration protocols for *S. halophila* should be optimized separately.

### Effect of cytokinin types (BAP, mT, KIN)

**Fig. 4:**
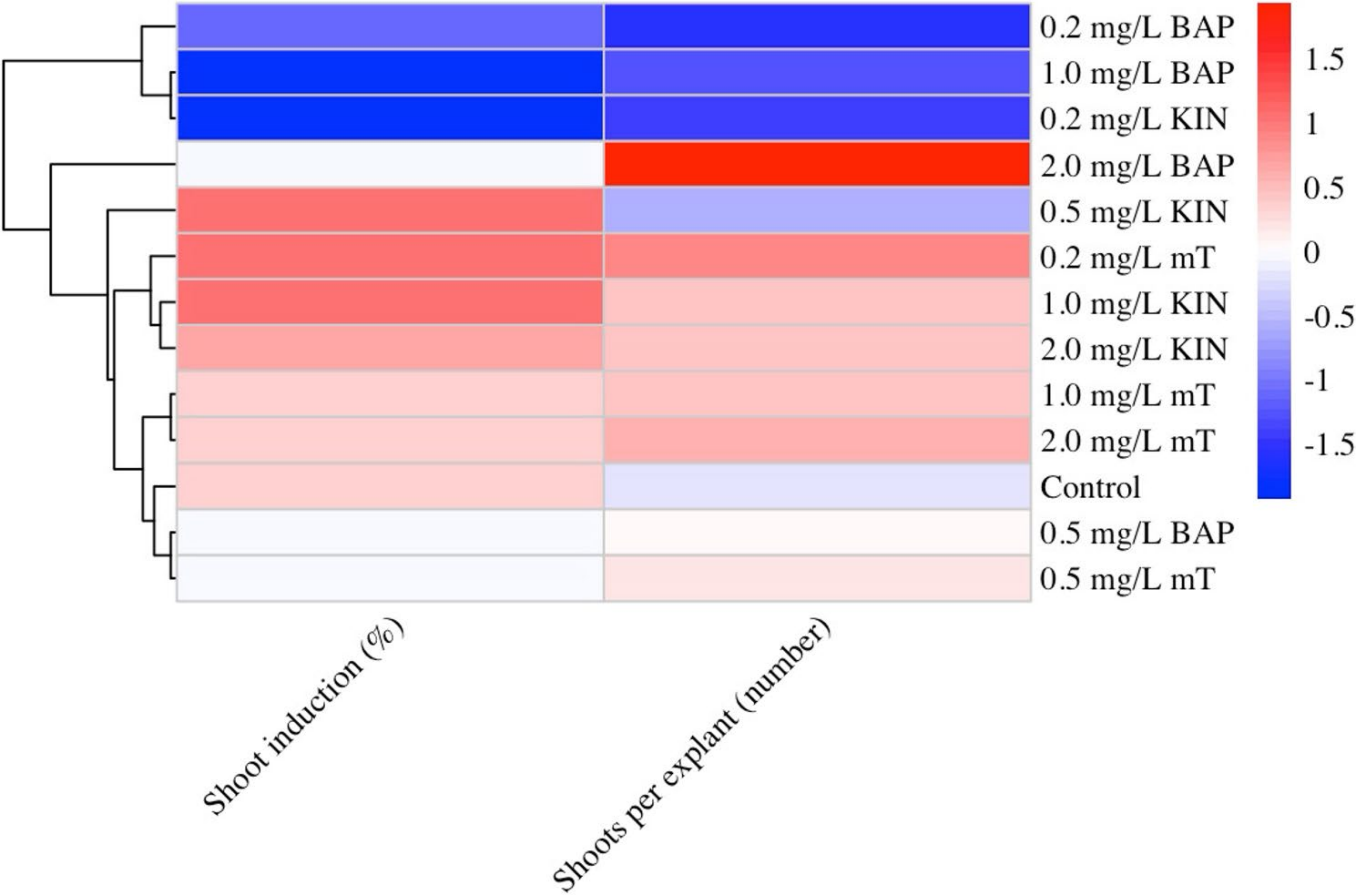
Heatmap visualization of the effects of cytokinin type and concentration on shoot induction percentage and mean shoot number per explant in *S. halophila* nodal explants. Color intensities denote response magnitude

The effects of different cytokinin types (BAP, mT, and KIN) and concentrations on shoot induction (%) and the number of shoots per explant in *S. halophila* nodal explants are presented in Table 5 and the heatmap (Fig. 4). According to the analysis of variance, both cytokinin type and concentration significantly influenced shoot induction (%) and shoot number per explant (*P* < 0.05). Moreover, the PGR × concentration interaction was also significant for both parameters (*P* < 0.05).

**Table 5:** Effects of different cytokinin types (BAP, mT, and KIN) and concentrations on shoot induction (%) and shoots per explant of *S. halophila*

(mg L⁻¹)	BAP		mT		KIN	
	Shoot induction (%)	Shoots explant⁻¹	Shoot induction (%)	Shoots explant⁻¹	Shoot induction (%)	Shoots explant⁻¹
0.0	90 ± 2.89ᵃᵇ	1.52 ± 0.07ᵃᶜ	90 ± 2.89ᵃᵇ	1.52 ± 0.07ᵃᶜ	90 ± 2.89ᵃᵇ	1.52 ± 0.07ᵃᶜ
0.2	70 ± 2.89ᵃᵇ	1.00 ± 0.00ᶜ	100 ± 0.00ᵃ	1.92 ± 0.04ᵃᵇ	60 ± 4.08ᵇ	1.05 ± 0.03ᵇᶜ
0.5	85 ± 4.79ᵃᵇ	1.60 ± 0.11ᵃᶜ	85 ± 4.79ᵃᵇ	1.65 ± 0.10ᵃᶜ	100 ± 0.00ᵃ	1.35 ± 0.07ᵇᶜ
1.0	60 ± 4.08ᵇ	1.10 ± 0.16ᵇᶜ	90 ± 5.00ᵃᵇ	1.75 ± 0.13ᵃᶜ	100 ± 0.00ᵃ	1.75 ± 0.13ᵃᶜ
2.0	85 ± 2.50ᵃᵇ	2.30 ± 0.06ᵃ	90 ± 2.89ᵃᵇ	1.80 ± 0.07ᵃᶜ	95 ± 2.50ᵃ	1.75 ± 0.09ᵃᶜ
Mean	78ᴮ	1.51	91ᴬ	1.73	89ᴬ	1.48

Values are mean ± SE (*n* = 4). Two-way ANOVA and Tukey’s HSD test (*P* ≤ 0.05) were used. Lowercase letters indicate significant differences among cytokinin × concentration interaction means; uppercase letters indicate main factor differences among cytokinin types

In BAP treatments, shoot induction rates ranged from 60.0% (1.0 mg L⁻¹) to 90.0% (control). The highest shoot number was obtained at 2.0 mg L⁻¹ BAP (2.30 ± 0.06 shoots per explant); however, induction at this concentration (85.0%) was lower compared with mT and KIN. mT applications yielded more consistent responses, with induction rates between 85.0% (0.5 mg L⁻¹) and 100.0% (0.2 mg L⁻¹). Particularly, 0.2 mg L⁻¹ mT achieved 100% induction with 1.92 ± 0.04 shoots per explant, representing the best outcome overall. At 1.0 mg L⁻¹ mT, 90% induction and 1.75 ± 0.13 shoots per explant were also recorded. For KIN, induction varied between 60.0% (0.2 mg L⁻¹) and 100.0% (0.5–1.0 mg L⁻¹), with shoot numbers ranging from 1.05 ± 0.03 to 1.75 ± 0.13.

On average, the highest shoot induction was recorded with mT (91%) and KIN (89%), while BAP was significantly lower (78%). Regarding shoot number per explant, mT yielded the highest mean (1.73). After four weeks of culture, the induced shoots reached an average length of 2.5–3.5 cm across treatments, and up to 3–4 cm under the optimal 0.2 mg L⁻¹ mT condition.

The heatmap (Fig. 4) provided an integrative overview of cytokinin × concentration combinations. Visual analysis revealed that mT, particularly at low to medium concentrations (0.2–1.0 mg L⁻¹), ensured both high induction (90–100%) and high shoot numbers (1.75–1.92). KIN was distinguished by consistently high induction rates (up to 95–100%), whereas BAP only enhanced shoot numbers at 2.0 mg L⁻¹ (2.30 shoots per explant) but remained comparatively limited in induction response.

The highest shoot induction rate in *S. halophila* nodal explants was obtained with mT applications, reaching 90–100% induction at low concentrations (0.2–1.0 mg L⁻¹). Consistently, the superiority of mT over BAP in terms of shoot induction and morphological quality has been reported in several *Salvia* species, including *S. sclarea* [22], *S. officinalis*, *S. fruticosa*, *S. tomentosa*, *S. pomifera*, and *S. ringens* [20], as well as *S. viridis* [119] and *S. bulleyana* [24]. These findings collectively confirm that mT has emerged as a promising alternative cytokinin for aromatic and medicinal plants in recent years.

KIN treatments also resulted in high induction rates (60–100%); however, the number of shoots per explant remained within the range of 1.35–1.75. Similar observations have been reported previously, indicating that although KIN exhibits a strong capacity for shoot initiation, its multiplication efficiency is lower than that of BAP or mT [20]. Indeed, in various *Salvia* species, KIN has been shown to be effective in initiating shoots but more limited in terms of shoot proliferation and the number of shoots produced, relative to mT and BAP [22, 24]. Therefore, in protocols targeting high multiplication efficiency, mT or BAP are generally more advantageous.

BAP treatments increased the number of shoots only at the highest concentration (2.0 mg L⁻¹), reaching 2.30 ± 0.06 shoots per explant, while the induction rate remained limited (85%). Similarly, in various *Salvia s*pecies, BAP at higher concentrations may enhance shoot number, but induction frequency and shoot quality are often restricted, and hyperhydricity as well as morphological abnormalities have been observed at elevated doses [25, 98, 100]. Alternative cytokinins such as mT and BAP derivatives have been shown to ensure more balanced and higher-quality multiplication across most species. Overall, the results highlight the potential of mT as a superior cytokinin for efficient and morphologically stable shoot induction in *S. halophila*, providing a basis for optimized micropropagation protocols.

### Rooting and acclimatization

**Fig. 5:**
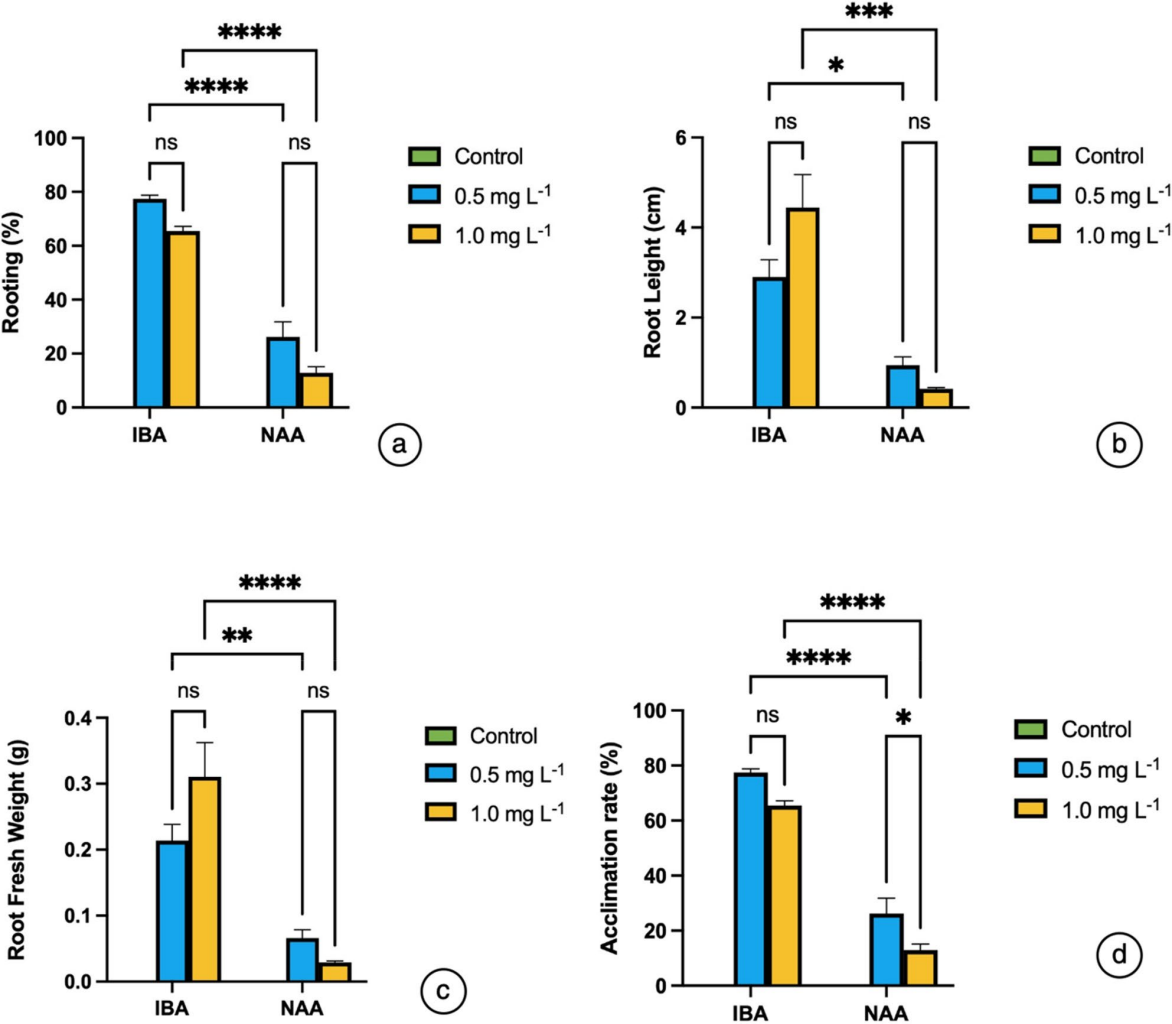
Effect of auxin type (IBA and NAA) and concentration on rooting and acclimatization of *S. halophila* regenerated shoots. **a** Rooting percentage; (**b**) Root length; (**c**) Root fresh weight; (**d**) Acclimatization rate. Values are mean ± SE (*n* = 4). Statistical differences were determined by two-way ANOVA followed by Tukey’s HSD test (*P ≤ 0.05, **P ≤ 0.01, ***P ≤ 0.001, ****P ≤ 0.0001)

The effects of different auxin types (IBA and NAA) and concentrations on rooting and acclimatization of regenerated shoots of *S. halophila* were systematically evaluated (Table S1). Two-way ANOVA revealed that both auxin type and concentration had statistically significant effects on all rooting parameters (rooting percentage, root length, root fresh weight, and acclimatization rate) (*P* < 0.05). The results demonstrated a clear superiority of IBA over NAA in terms of root induction and acclimatization success. At 0.5 mg L⁻¹ IBA, the highest rooting induction (66.22 ± 1.23%) and acclimatization efficiency (77.47 ± 0.48%) were achieved, whereas at 1.0 mg L⁻¹ IBA the rooting percentage declined (40.00 ± 2.04%), but root elongation (4.44 ± 0.26 cm) and biomass accumulation (0.31 ± 0.018 g) reached their maximum levels. These findings indicate that lower concentrations of IBA are more effective for root induction, while higher concentrations promote root elongation and biomass production. In contrast, NAA exhibited limited effects across all parameters, resulting in low rooting percentages (25.00 ± 1.02%), short roots (0.94 ± 0.06 cm), reduced fresh weight (0.066 ± 0.005 g), and poor acclimatization success (26.20 ± 1.97%). This poor performance of NAA may be attributed to its rapid metabolic degradation and the tendency of higher NAA doses to induce cellular stress or inhibit organized root formation. Overall means also confirmed the statistically significant superiority of IBA over NAA for all traits (*P* < 0.05). Graphical analysis (Fig. 5) further supported the tabulated results, statistically confirming the superiority of IBA over NAA in rooting and acclimatization parameters (*P ≤ 0.05, **P ≤ 0.01, ***P ≤ 0.001, ****P ≤ 0.0001).

Consistently, IBA applications provided marked advantages over NAA with respect to root induction, root development, and acclimatization success. The superiority of IBA in root induction is primarily associated with its biochemical behavior within auxin metabolism and transport. IBA is a more stable auxin form that is oxidized more slowly than IAA, allowing optimal auxin levels required for root initiation to be maintained for a longer period [120, 121]. Additionally, IBA contributes to the active auxin pool through its conversion to IAA in peroxisomes, a process that promotes cell division and differentiation in the root meristem [122]. IBA also enhances polar auxin transport, facilitating the establishment of an auxin gradient essential for adventitious root primordium initiation [120, 122]. Consequently, IBA generally results in higher rooting percentages, greater root numbers, and stronger root elongation than NAA [123]. In contrast, NAA tends to accumulate more rapidly in tissues and may cause toxicity at elevated concentrations, inhibiting root elongation [124]. Although low concentrations of NAA may support root induction, its inhibitory effects at higher levels can restrict optimal root development [125]. Lower concentrations induced root induction, whereas higher concentrations were more effective for root growth and biomass accumulation. Conversely, NAA displayed limited performance under all treatments and was inadequate in terms of acclimatization success.

Comparable results have been reported in micropropagation studies of other *Salvia* species. For instance, IBA was shown to improve rooting efficiency and acclimatization in *S. tomentosa* [25] and *S. officinalis* [20], while *S. hispanica* [126] exhibited high rooting success under IBA treatment. Nevertheless, in some species NAA has been found to be effective at specific concentrations. In *S. sclarea* [22] and *S. miltiorrhiza* [26], low levels of NAA supported successful rooting and high acclimatization rates. Furthermore, optimization studies in *S. macrosiphon* revealed that a combination of IBA and NAA could enhance rooting responses [60]. These variations indicate that rooting responses are species-dependent, yet the general trend highlights IBA as the more effective auxin for root induction and development across the genus. Overall, the present findings confirm that IBA is the most effective auxin for rooting and acclimatization in *S. halophila*, with 0.5 mg L⁻¹ providing the optimum results in terms of both root induction and successful adaptation to *ex vitro* conditions. Similar findings have been reported for related *Salvia* species, supporting the conclusion that NAA has only a limited effect on rooting performance in this species.

**Fig. 6:**
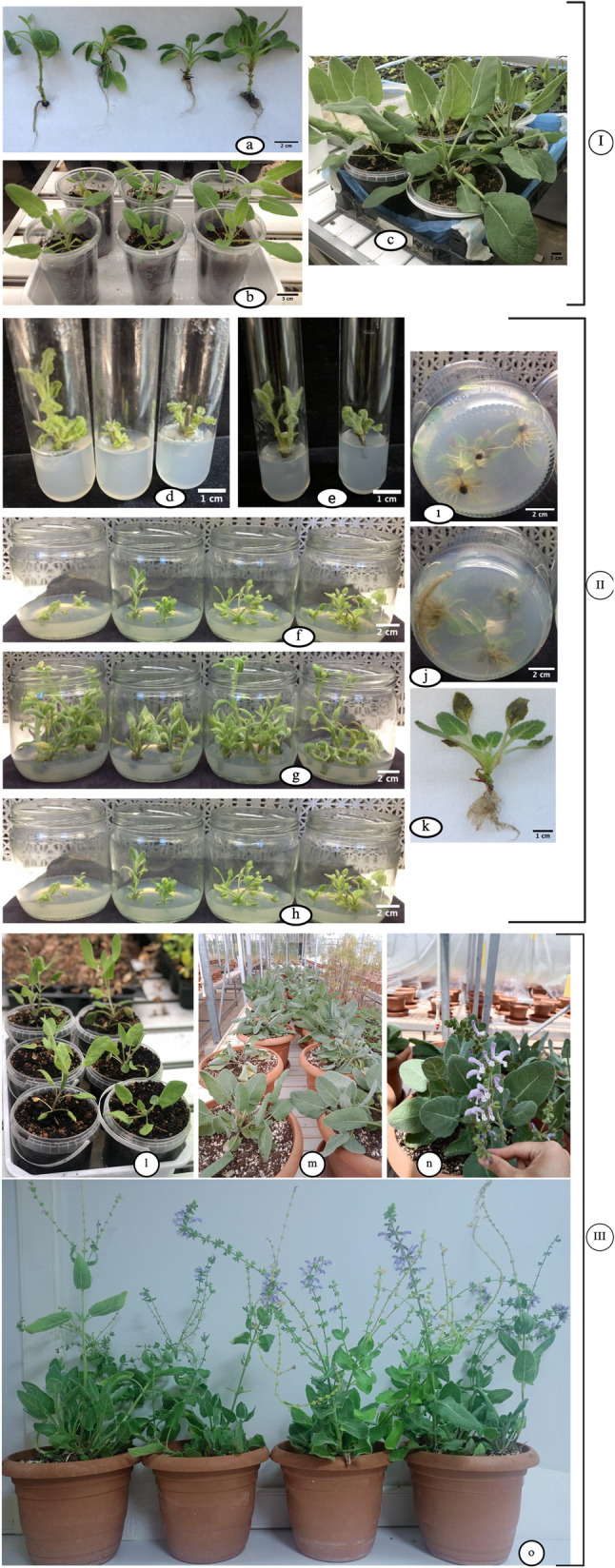
Stage I–II: In vitro germination, early acclimatization, and shoot regeneration of *S. halophila*. Stage I (In vitro germination and early acclimatization): (**a**) Seedlings obtained after in vitro germination on GA₃-supplemented media (**b**) One-month-old plantlets transferred to sterile substrate for early acclimatization (**c**) Three-month-old, acclimatized plantlets with well-developed morphology. Stage II (Shoot regeneration, proliferation and rooting): (**d–e**) Shoot regeneration from nodal segments subjected to different surface sterilization protocols (**f**) Shoot proliferation on MS medium supplemented with BAP, (**g**) mT, and (**h**) KIN after 3 weeks of culture (**i**) Root induction on regenerated shoots under NAA treatment and (**j**) under IBA treatment (**k**) Fully rooted plantlet. Stage III: Ex vitro acclimatization and greenhouse development of S. halophila. (**l**) One-month-old, acclimatized plants transferred to plastic pots (**m**) Two-month-old plantlets established in the greenhouse (**n**-**o**) Four-month-old greenhouse-grown plants reaching the flowering stage

A schematic overview of the optimized in vitro propagation workflow of *S. halophila*, from seed germination through acclimatization and flowering, is presented in Fig. 6.

### Phenolic and antioxidant contents of micropropagated plantlets

**Fig. 7:**
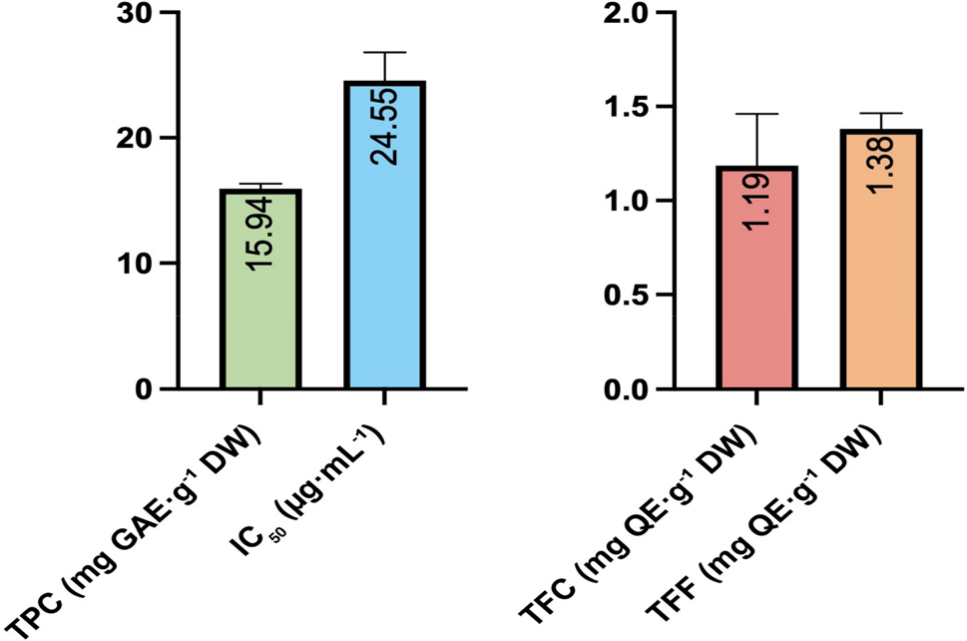
Phenolic and antioxidant contents of micropropagated *S. halophila* plantlets obtained under optimized conditions. Values are mean ± SE (*n* = 3)

Under the optimized micropropagation conditions (MS medium supplemented with 0.2 mg L⁻¹ mT for shoot induction and 0.5 mg L⁻¹ IBA for rooting), acclimatized *S. halophila* plantlets were evaluated for their biochemical composition (Table S2, Fig. 7). Total phenolic content (TPC) was determined as 15.94 ± 0.24 mg GAE g⁻¹ DW, total flavonoid content (TFC) as 1.19 ± 0.16 mg QE g⁻¹ DW, and total flavonol content (TFF) as 1.38 ± 0.05 mg QE g⁻¹ DW. Antioxidant capacity, measured by the DPPH assay, yielded an IC₅₀ value of 24.55 ± 1.30 µg mL⁻¹. To accurately interpret these biochemical results, it should be noted that seed-derived seedlings and micropropagated plantlets represent distinct physiological stages and have experienced different culture conditions. Therefore, differences in TPC, TFC, TFF, and antioxidant capacity may partly reflect developmental and environmental disparities rather than treatment effects alone. For this reason, direct comparisons between these two groups should be interpreted cautiously, and the biochemical profiles of each group are considered within their own experimental context.

Within this framework, micropropagated plantlets exhibited a distinct biochemical pattern compared to seedlings obtained under different media and GA₃ treatments. Phenolic content was approximately three-fold higher, flavonoid levels were comparatively lower, and flavonols showed a slight increase. Antioxidant capacity was also stronger, with IC₅₀ values nearly halved relative to seed-derived plantlets. These differences likely reflect the combined influence of developmental stage, in vitro growth conditions, and metabolic reprogramming associated with micropropagation.

Several studies on other *Salvia* species also report shifts in secondary metabolism under in vitro propagation. For instance, in *S. miltiorrhiza*, micropropagated plantlets were reported to accumulate higher phenolic and flavonoid levels and to exhibit stronger antioxidant activity compared to seedlings of the same age [26]. Likewise, elicitor applications (nano-TiO₂, methyl jasmonate) in *S. tebesana* have been shown to boost secondary metabolite accumulation, particularly rosmarinic acid, quercetin, and rutin [127]. In *S. sclarea*, flavonoid biosynthesis was found to vary with the type of cytokinin applied [22, 23], while in vitro-derived *S. officinalis* demonstrated strong radical-scavenging activity in methanolic extracts [128]. Taken together, these findings indicate that in vitro propagation commonly alters secondary metabolite profiles across *Salvia* species, consistent with the trends observed in the present study.

A comparison with wild-collected *S. halophila* further illustrates these metabolic differences. As reported in [5] ethanol extracts of wild plants contained lower phenolic levels and exhibited weaker antioxidant activity, whereas micropropagated plantlets in the present study showed higher phenolic accumulation and stronger radical-scavenging potential. On the other hand, Koşar et al. [6] documented considerably higher phenolic content in 50% methanolic extracts of wild populations, emphasizing that solvent polarity and extraction fractionation strongly influence phytochemical yield. Taken together, these observations indicate that in vitro–derived *S. halophila* plantlets display a metabolic profile that differs from that of wild plants, shaped by both culture conditions and extraction methodology, rather than representing a direct superiority.

## Conclusions

This study developed an effective and stage-specific optimized in vitro propagation protocol for the endangered endemic sage *S. halophila*. At each experimental phase, the highest-performing treatments were identified, and these outcomes were integrated into a stepwise optimization strategy.

During the germination stage, the highest success was achieved through in vitro GA₃ supplementation; WPM medium containing 0.5–1.0 mg L⁻¹ GA₃ produced the maximum germination rate of 20%, outperforming all conventional priming attempts. For culture establishment, the combined sterilization treatment of HgCl₂ and 2 mg L⁻¹ PPM™ resulted in the most reliable initiation, reducing fungal contamination to 4.4% and enabling 73% healthy shoot survival. In shoot induction, MS medium—particularly with low BAP levels and 0.2 mg L⁻¹ mT—provided the highest induction success (up to 100%) and superior multiplication efficiency. Among cytokinins, meta-topolin (mT) was the most effective regulator, offering the best balance between induction and proliferation. For rooting, 0.5 mg L⁻¹ IBA yielded the highest rooting percentage (66%) and acclimatization success (77%), whereas 1.0 mg L⁻¹ IBA maximized root elongation and biomass, demonstrating a clear concentration-dependent shift from induction to elongation. Biochemical profiling of plantlets obtained under fully optimized conditions further validated the protocol’s efficiency. Optimized plantlets accumulated 15.94 mg GAE g⁻¹ DW total phenolics and exhibited strong antioxidant activity with an IC₅₀ of 24.55 µg mL⁻¹, representing the highest biochemical performance recorded in the study.

Overall, these findings confirm the effectiveness of a stage-based and optimization-driven micropropagation strategy for *S. halophila*. This stage-based, optimization-driven workflow provides a scalable and reliable strategy for *ex situ* conservation, standardized metabolite production, and potential commercial propagation of *S. halophila*. Additionally, this optimized protocol offers a strong foundation for future studies on genetic stability, elicitor-enhanced metabolite production, and field-level performance of regenerated plants.

## Supplementary Information

Supplementary Material 1.

## Data Availability

All data are presented in this article and in Supplementary Table S1, Table S2. Additional raw data are available from the author upon reasonable request.

## Abbreviations

ANOVA: Analysis of Variance
AS: Acclimatization Success
BAP: 6-Benzylaminopurine
B5: Gamborg B5 medium
DPPH: 2,2-Diphenyl-1-picrylhydrazyl
DW: Dry Weight
GA₃: Gibberellic Acid
GAE: Gallic Acid Equivalent
HgCl₂: Mercuric Chloride
IBA: Indole-3-Butyric Acid
IC₅₀: Half Maximal Inhibitory Concentration
KIN: Kinetin
mT: meta-Topolin
MS: Murashige and Skoog medium
NAA: α-Naphthaleneacetic Acid
NaOCl: Sodium Hypochlorite
PCA: Principal Component Analysis
PPM™: Plant Preservative Mixture™
PGR: Plant Growth Regulator
QE: Quercetin Equivalent
RL: Root Length
RFW: Root Fresh Weight
TPC: Total Phenolic Content
TFC: Total Flavonoid Content
TFF: Total Flavonol Content
WPM: Woody Plant Medium
