# Supplementary material for: Development of an efficient in vitro micropropagation and biochemical profiling of Salvia halophila, an endemic Turkish sage
Source: BMC Plant Biol. 2026 Jan 19;26:289. doi: 10.1186/s12870-026-08152-2 (PMC12896189; doi:10.1186/s12870-026-08152-2)
Supplement: Supplementary file 1 — Supplementary Material 1. [file 12870_2026_8152_MOESM1_ESM.zip › Supplementary Materials.docx]

**Supplementary Materials**

**Table S1.** Effect of auxin type (IBA and NAA) and concentration on rooting parameters and acclimatization success of *in vitro* regenerated shoots^1^

| **(mg L⁻¹)** | **IBA** | | | | **NAA** | | | |
| --- | --- | --- | --- | --- | --- | --- | --- | --- |
|  | **Rooting (%)** | **RL (cm)** | **RFW (gr)** | **AS (%)** | **Rooting (%)** | **RL (cm)** | **RFW (gr)** | **AS (%)** |
| **0.0** | 0 ± 0^c^ | 0 ± 0^b^ | 0 ± 0^b^ | 0 ± 0^d^ | 0 ± 0^c^ | 0 ± 0^b^ | 0 ± 0^b^ | 0 ± 0^d^ |
| **0.5** | 66.22 ± 1.23ᵃ | 2.90±0.13^a^ | 0.22± 0.009^a^ | 77.47± 0.48^a^ | 25.00 ± 1.02^b^ | 0.94± 0.06^b^ | 0.066± 0.005^b^ | 26.20± 1.97^b^ |
| **1.0** | 40.00 ± 2.04^a^ | 4.44± 0.26^a^ | 0.31± 0.018^a^ | 65.43± 0.61^a^ | 25.00 ± 1.02ᵇ | 0.42± 0.01^b^ | 0.029 ± 0.001^b^ | 12.87± 0.79^c^ |
| **mean** | **35.40ᴬ** | **2.44^A^** | **0.018^A^** | **47.63^A^** | **16.66ᴮ** | **0.45^B^** | **0.032^B^** | **13.02^B^** |

^1^Values are presented as mean ± SE (n = 4). Different superscript letters within a column indicate significant differences according to Tukey’s test (P ≤ 0.05). Capital letters indicate significant differences between auxin types (IBA vs. NAA). PGR × Conc.: rooting (%)^**^;RL^**^; RFW^**^;AS^**;^ PGR **:** rooting (%)^**^;RL^**^; RFW^**^;AS^**^ ;* P < 0.05; ** P < 0.01.

**Table S2.** Phenolic and antioxidant profile of *S. halophila* plantlets obtained under optimized micropropagation conditions.

| **Parameter** | **Value (Mean ± SE)** |
| --- | --- |
| TPC (mg GAE·g⁻¹ DW) | 15.94 ± 0.24 |
| TFC (mg QE·g⁻¹ DW) | 1.19 ± 0.16 |
| TFF (mg QE·g⁻¹ DW) | 1.38 ± 0.05 |
| IC₅₀ (µg·mL⁻¹) | 24.55 ± 1.30 |
